# High-altitude de-acclimatization and long-term immune suppression: the role of Nrf2 in Treg function targeting 3PM

**DOI:** 10.1007/s13167-026-00442-x

**Published:** 2026-03-04

**Authors:** Yuxin Wang, Zhijie Bai, Jiamiao Li, Jinfeng Liu, Yunan Zhang, Xizheng Wang, Lei Zhou, Haoyu Ni, Pan Shen, Ningning Wang, Zhexin Ni, Chaoji Huangfu, Wei Zhou

**Affiliations:** 1 Beijing Institute of Radiation Medicine, Beijing, China; 2 Hospital of Chengdu University of Traditional Chinese Medicine, Chengdu, China; 3 Chengdu University of Traditional Chinese Medicine, Chengdu, China; 4 School of Chinese Materia Medica, Tianjin University of Traditional Chinese Medicine, Tianjin, China; 5 College of Chinese Materia Medica, Yunnan University of Chinese Medicine, Kunming, China; 6 School of Life Sciences, Beijing University of Chinese Medicine, Beijing, 102488 China

**Keywords:** Predictive preventive personalized medicine (PPPM / 3PM), High altitude de-acclimatization(HADA), Health risk stratification, Oxidative stress, Individualized profile, Lifestyle and nutritional intervention, Immune cell, Treg, Immunity suppression, Nrf2, RNA-seq, ATAC-seq, Flow cytometry

## Abstract

**Background:**

High-altitude de-acclimatization (HADA) is accompanied by a complex spectrum of long-term physiological and functional remodeling processes, potentially affecting long-term health outcomes. Existing studies on HADA have predominantly focused on cardiovascular and nervous system changes. However, the immune system—an essential regulator of disease susceptibility, inter-individual variability, and long-term health risks—remains insufficiently investigated in the context of HADA.

**Aims:**

Given the central role of immune regulation in maintaining systemic homeostasis and determining individual health trajectories, elucidating immune alterations associated with HADA is essential. The present study aims to characterize immune system remodeling during HADA with particular emphasis on its functional outcomes and mechanisms. By addressing these scientific questions, this study seeks to provide an immune system perspective for the health maintenance of HADA individuals, promoting paradigm shift from reactive medical services toward predictive, preventive, and personalized medicine (3PM).

**Methods:**

Peripheral blood was collected from both human cohorts and mice models while other immune organs including spleen, thymus and bone marrow were obtained from mice models. Proportions of immune cell populations in peripheral blood and other immune organs were analyzed using flow cytometry. The immune-suppressive functions of Tregs were determined by in vitro co-culture with CD8^+^ T cells. Transcriptomic and chromatin-accessibility feature induced by HADA were obtained through RNA-seq and ATAC-seq. The functional validation of HADA target gene was performed using specific agonist during in vitro co-culture system.

**Results:**

HADA perturbed the proportions of immune populations in multiple immune site. Both data from human and mice showed increased regulatory T cells (Tregs) and enhanced immune-suppressive function in the peripheral blood. As a consequence, these Tregs mediated long-term immune suppression and compromised the immunity against tumor cells. Multi-omic analyses predicted Nrf2 as the key mediator of molecular alterations in Tregs caused by HADA, which was further confirmed by the functional assay.

**Conclusion and expert recommendation:**

This study advances high-altitude medicine by demonstrating that HADA induces long-lasting immunosuppressive effects through Nrf2-mediated Treg remodeling, with important implications for immune homeostasis and long-term health risks. These findings highlight the role of the immune system, particularly Tregs, in HADA-induced health impairments and identify Nrf2 as a potential therapeutic target. Moreover, immune biomarkers—especially Treg phenotypes and Nrf2 activity—may serve as promising candidates for risk stratification and predictive diagnostics in populations transitioning between high- and low-altitude environments. Preventive strategies should prioritize immune-informed recovery protocols, oxidative stress modulation, and lifestyle or nutritional interventions tailored to individual immune profiles.

**Supplementary Information:**

The online version contains supplementary material available at 10.1007/s13167-026-00442-x.

## Introduction

Exposure to high altitude imposes substantial physiological stress on the human body due to the hypobaric hypoxic environment,necessitating adaptive responses to maintain homeostasis and functional capacity [1]. While extensive research has focused on high-altitude acclimatization, considerably less attention has been given to high-altitude de-acclimatization (HADA), the process of re-adaptation that occurs upon returning to low-altitude environments after prolonged high-altitude residence [2]. Health problems concerning this process were briefly reported and discussed in early studies on high-altitude biology dating back to the early 1900s, but received limited attention at the time [3]. In China, the incidence of high-altitude de-acclimatization syndrome (HADAS) has been reported to approach 85%, with even higher rates observed among individuals with longer durations of high-altitude exposure or engagement in physically demanding manual labor at high altitude. Recently, public health research has increasingly emphasized the need to recognize HADA-related disorders as a significant health and safety concern [4]. The predictive, preventive, and personalized medicine (PPPM) paradigm has been widely recognized as a valuable framework for managing chronic diseases, such as atherosclerosis, by integrating individualized risk prediction with targeted preventive strategies and personalized healthcare delivery [5]. However, despite its success in chronic disease management, the application of the PPPM framework to high-altitude de-acclimatization—particularly its immune-related long-term consequences—remains largely unexplored.

## HADA-induced immune perturbations require more attention

Current research indicates that the immune system undergoes substantial reorganization during high-altitude acclimatization [6, 7]. Studies combining single-cell transcriptomics and metabolomics have revealed that hypoxic exposure triggers dynamic changes in immune cell populations involving myeloid cells with downregulated inflammatory responses and CD8^+^ T cells and γδ T cells with enhanced effector functions [8]. At the molecular level, these adaptations are mediated through hypoxia-inducible factors (HIFs) [9] that regulate cellular metabolism, particularly shifting immune cells toward glycolytic pathways. Multi-omics analyses further demonstrate significant alterations in plasma metabolites, including increased glutamine and fatty acids, which support immune cell energy requirements in oxygen-limited environments [10]. Additionally, animal studies have identified key signaling pathways, including JAK-STAT and NOD-like receptor pathways, as critical regulators of immune adaptation to hypoxia [11].

The HADA process, often accompanied by a constellation of clinical symptoms including dizziness, excessive sleepiness, palpitations, chest tightness, and sleep disturbances, exerts long-term effects on health [12, 13]. The immunological changes observed during high-altitude exposure do not immediately revert upon returning to plains. Short-term studies (up to 15 days post-return) showed persistent alterations in immune parameters, including increased CD8^+^ T cell and natural killer (NK) cell percentages, decreased CD4^+^/CD8^+^ ratios, and elevated cytokine-induced killer cell levels [14]. Longer exposures to high altitude (≥ 1 year) appeared to induce more sustained changes. Longitudinal human studies have documented systemic immune disturbances lasting up to 90 days after returning from high altitude, with the development of altitude-specific immune scores that correlate with hypoxia degree, hematopoietic function, and liver enzyme changes [15]. Within the PPPM framework, more attention should be given to the long-term immune alterations induced by HADA and the underlying mechanisms, underscoring the need for predictive biomarkers and targeted strategies to promote immune homeostasis.

### Tregs exerts important functions for immune homeostasis

Regulatory T cells (Treg), a specialized subset of CD4⁺ T cells with potent immunosuppressive properties, play a central role in maintaining immune homeostasis and self-tolerance [16]. Beyond suppressing conventional effector T cells, Tregs exert broad immunomodulatory functions by regulating B cells, natural killer (NK) cells, and various non-lymphoid cell populations, including those residing in adipose tissue [17]. The transcription factor forkhead box P3 (Foxp3) is the lineage-defining regulator of Treg cells and is essential for their development, stability, and suppressive function. Accordingly, Foxp3⁺ Tregs are critical regulators of immune balance and contributors to immune homeostasis and self-tolerance [18]. Maintaining an appropriate Treg equilibrium is crucial for immune resilience: excessive Treg activity may predispose individuals to immunodeficiency, persistent infections, and tumor progression, whereas insufficient Treg function can increase susceptibility to autoimmunity, immune-mediated tissue damage, and impaired antimicrobial defense [19]. Numerous studies have focused on the effects of hypoxia on the differentiation and function of Tregs. It was reported that hypoxia widely regulated the balance of Treg/Th17 cell differentiation under specific pathological conditions. In a recently published work, hypoxia remarkedly downregulated the differentiation from CD4+ naïve T cells into Tregs and enhanced the generation of Th17 cells in an in vitro differentiation system.Regrettably, the current research on hypoxia and Treg has not yet addressed the changes in immunosuppression function of Tregs. The research on HADA effects on Tregs, either on quantitative or the functional level, is even a complete blank.

## The working hypothesis and aims

We hypothesized that the consistent increase in Tregs observed in the peripheral blood of both the HADA cohort and corresponding animal models is accompanied by significant alterations in immune function and long-term health outcomes. We further proposed that these changes are regulated by specific molecular mechanisms, particularly transcription factors. To test this hypothesis, we systematically assessed the function of Treg and performed transcriptomic profiling of Tregs using RNA sequencing, together with chromatin accessibility analysis of peripheral white blood cells using ATAC sequencing, to dissect the molecular mechanisms underlying Treg expansion and functional reprogramming. Ultimately, this study aims to identify key regulatory molecules that may enable targeted preventive or therapeutic interventions against HADA-associated immune disturbance, thereby supporting proactive immune risk stratification and personalized intervention strategies.

## Methods and materials

### Human participants and mouse handling

A total of 32 male participants aged 25–40 years were enrolled in this study. All HADA participants were long-term high-altitude migrants (residing at above 3500 m for more than 5 years) who had relocated to low-altitude areas (below 500 meters) for more than 0.5 years. None of the participants had any primary or severe diseases. The control group consisted of 20 healthy male plains dwellers (average elevation: 500 m) aged 25–35 years with no history of high-altitude residence, who underwent routine physical examinations at the hospital during the same period. Peripheral blood was collected from each participant before breakfast for flow cytometry analysis, complete blood count, and blood biochemical tests. The study was conducted with the approval of the Ethics Committee of the Beijing Institute of Radiation Medicine (approval number: AF/SC-08/02.153). All participants provided written informed consent in accordance with the Declaration of Helsinki.

The 8-week old male C57BL/6j mice were purchased from GemPharmatech Co., Ltd. (Beijing, China). All mice were housed in a specific pathogen-free (SPF) facility at the Academy of Military Medical Sciences. A hypobaric hypoxia chamber was used to simulate high-altitude conditions via a vacuum pump to create a low-pressure, low-oxygen environment, with an internal pressure sensor to accurately mimic specific altitudes. In detail, an altitude of 6,000 m was simulated, with the chamber temperature maintained at 25 °C and relative humidity at approximately 60%. Twenty-eight days after being fed in the chamber, mice were transferred to normoxic conditions for HADA model construction. Mice in all groups had continuous access to food and water throughout the exposure period. The Animal Care and Use Guidelines received approval from the Beijing Institute of Radiation Medicine, with the approval number IACUC-DWZX-2025-509.

## Enzyme linked immunosorbent assay

Prior to quantitative analysis, preliminary optimization experiments were performed to determine the appropriate dilution factors for each analyte in human serum and murine plasma samples. Subsequently, cytokine concentrations were measured in strict accordance with the manufacturers’ instructions using commercially available ELISA kits, including IFN-γ (JONLNBIO, JL12152-96T, China), IL-1β (JONLNBIO, JL13662-96T, China), TNF-α (JONLNBIO, JL10208-96T, China), TGF-β (JONLNBIO, JL20082-96T, China), and IL-6 (JONLNBIO, JL14113-96T, China).

### Cytokines level detection

Mouse plasma samples were analyzed undiluted on the Luminex X200 instrument (Austin, TX, USA) following the manufacturer’s protocol for the *Th1/Th2/Th9/Th17/Th22/Treg Cytokine 17-Plex Mouse Panel* (LAIZEE BIOTECH, EPX170-26087-901, Shanghai, China). Splenic and other tissue homogenates were quantified using the bicinchoninic acid (BCA) protein assay and normalized to equivalent protein concentrations prior to multiplex analysis.

## Flow cytometric analysis

Following erythrocyte lysis using Red Blood Cell Lysis Buffer (Solarbio, R1010, Beijing, China), whole blood samples were washed with phosphate-buffered saline (PBS) and resuspended in 100 µL PBS supplemented with 2% fetal bovine serum (FBS). Human peripheral blood immunophenotyping was performed according to established protocols as previously described [20]. 7-AAD (BD Biosciences, Franklin Lakes, NJ, USA) viability dye was employed to discriminate live/dead cell populations. For intracellular Foxp3 detection, cells underwent surface marker staining followed by fixation and permeabilization using the Foxp3/Transcription Factor Staining Kit (eBioscience, 00-5523-00, San Diego, CA, USA), then incubated with anti-Foxp3 monoclonal antibody (eBioscience, 12-5773-82) for 30 min at 4 °C under light-protected conditions. In Th1/Th2/Th17 subset analysis, cells were preconditioned in cell stimulation cocktail (eBioscience, 00-4970-93) -containing complete medium for 5–16 h prior to antibody labeling. To assess cellular proliferation, BrdU (10 mg/mL in PBS) was administered intraperitoneally at 1.2 mg per 25 g body weight 40 min prior to euthanasia. Peripheral blood and spleen samples were collected post-euthanasia and processed into single-cell suspensions through mechanical dissociation. Following the aforementioned staining protocols, cells were analyzed using a flow cytometer (BD FACSAria III) equipped with 405 nm, 488 nm, and 640 nm laser lines. Acquired data were subsequently processed through FlowJo software (v10.8.1) for advanced population gating and statistical analysis. The gating strategy [21] is shown in Supplementary Figure 1A.

## Immune suppressing function assay

Commenced with overnight coating of 96-well plates using phosphate-buffered saline (PBS) containing 2 µg/mL CD3 antibody (BioLegend ,100339, San Diego, CA, USA) at 4 °C under sterile conditions. Fresh spleens from naive C57BL/6 mice were subsequently processed into single-cell suspensions through mechanical dissociation and 70-µm filtration. CD8 + T lymphocytes were isolated by flow cytometry. Purified CD8^+^ T cells were resuspended at 1 × 10^6^ cells/mL in complete RPMI-1640 medium supplemented with CD28 antibody (2 µg/mL, Biolegend,102115). After removing the coating solution and performing triple PBS wash steps, CD8^+^ T cell aliquots were introduced into the antibody-precoated wells. Parallel processing involved fluorescence-activated cell sorting (FACS) of regulatory T cells (Tregs, defined as CD4^+^CD25^+^) from deacclimatized counterparts (14-day normoxic recovery). These Treg populations were adjusted to matching densities (1 × 10^6^ cells/mL) before being co-cultured with CD8^+^ T effectors at progressive ratios (1:2) in a humidified 37 °C incubator with 5% CO_2_ atmosphere. Following 72-hour incubation, the proliferation of CD8^+^ T cells was detected by flow cytometry.

Peripheral blood Treg cells were isolated from mice by flow cytometry. The purified Treg cells were pretreated with Bardoxolone methyl (MCE, HY-13324, Princeton, NJ, USA), a specific Nrf2 agonist, at the indicated concentration at 37 °C. After pretreatment, Treg cells were washed and co-cultured with CFSE-labeled (Thermo Fisher Scientific, Waltham, MA, USA), anti-CD3/CD28–stimulated CD8⁺ T cells in complete RPMI-1640 medium supplemented with 10% FBS. The co-cultures were maintained for 72 h at 37 °C in a humidified incubator with 5% CO₂. Following incubation, the proliferation of CD8⁺ T cells was evaluated by flow cytometry. Data were analyzed using FlowJo software (version 10.8.1). The concentration of IFN-γ in the supernatant was analyzed by ELISA.

### cDNA synthesis and quantitative PCR

Total RNA was extracted from sorted mouse peripheral blood cells, and cDNA was synthesized using the NEB low-input cDNA synthesis kit (New England Biolabs, E6420, Ipswich, MA, USA) according to the manufacturer’s instructions. Quantitative PCR (qPCR) was subsequently performed using the PerfectStart Green qPCR SuperMix (TransGen Biotech, AQ601-04-V2, Beijing, China) following the standard protocol provided by the manufacturer. Gene-specific primers were added to the reaction mixture, and amplification was carried out on a real-time PCR system under standard cycling conditions.

### RNA-seq and ATAC-seq of peripheral blood Treg

Peripheral blood Treg cells were sorted using flow cytometry. Due to the limited number of cells, cDNA library construction was performed using the NEBNext^®^ Single Cell/Low Input cDNA Synthesis & Amplification Module (NEB, E6421L, E7805, E6440) according to the manufacturer’s instructions. The libraries were kept at low temperature during transportation and sent to Novogene Co., Ltd. (Tianjin, China) for quality control and RNA sequencing following standard protocols.

Peripheral blood samples were subjected to red blood cell lysis, followed by two washes with PBS. Approximately 50,000 leukocytes were used for each ATAC-seq experiment. Cells were lysed on ice using a lysis buffer containing 10 mM Tris-HCl (pH 7.4), 10 mM NaCl, 3 mM MgCl₂, and 0.1% IGEPAL CA-630 to release nuclei. The nuclear pellets were resuspended in a transposition reaction mix containing Tn5 transposase and incubated at 37 °C for 30 min. Adapter 1 and Adapter 2 were added in equimolar amounts, and the libraries were amplified by PCR using the ATAC-seq_Novogene Kit (developed in-house by Novogene).

Following PCR amplification, libraries were purified with AMPure XP beads and quantified using a Qubit fluorometer. Paired-end sequencing (150 bp) was performed on the Illumina NovaSeq platform.

### Bio-informatic analyses

Raw sequencing data from both RNA-seq and ATAC-seq experiments were processed through a standardized bioinformatics pipeline to generate quantitative matrices. For RNA-seq, raw FASTQ files were first subjected to quality control using FastQC, followed by adapter trimming and low-quality read removal with Trimmomatic. High-quality reads were then aligned to the reference genome (GRCm39) using the STAR aligner. Gene expression quantification was performed using featureCounts, generating a count matrix where rows represent genes and columns represent samples. For ATAC-seq data, raw reads were similarly quality controlled with FastQC and trimmed using Trimmomatic. Cleaned reads were aligned to the reference genome using Bowtie2, with duplicate reads marked and removed using Picard Tools. Peak calling was executed with MACS2 to identify open chromatin regions, and a count matrix of peak intensities across samples was generated using tools like featureCounts or BedTools. Finally, both gene expression and chromatin accessibility matrices were normalized and prepared for downstream analyses.

For RNA-seq data, differential gene expression analysis was performed on the normalized gene expression matrix. Using the R package DESeq2 (version 1.42.0), genes with statistically significant differences between comparison groups were identified based on parameters such as absolute log2 fold change (|log2FC|) > 1 and adjusted *p* value < 0.05. For functional enrichment analysis, the list of differentially expressed genes (DEGs) was subsequently subjected to Gene Ontology (GO) pathway analyses using clusterProfiler (version 4.8.3). Terms and pathways with an adjusted *p* value < 0.05 were considered significantly enriched. Visualization of results was carried out using dot plots, bar plots, or enrichment maps to illustrate the key biological processes, molecular functions, cellular components, and signaling pathways perturbed across experimental conditions.

Clinical trial number: not applicable.

## Results

### Peripheral immune cells are perturbed during HADA phase

**Fig. 1:**
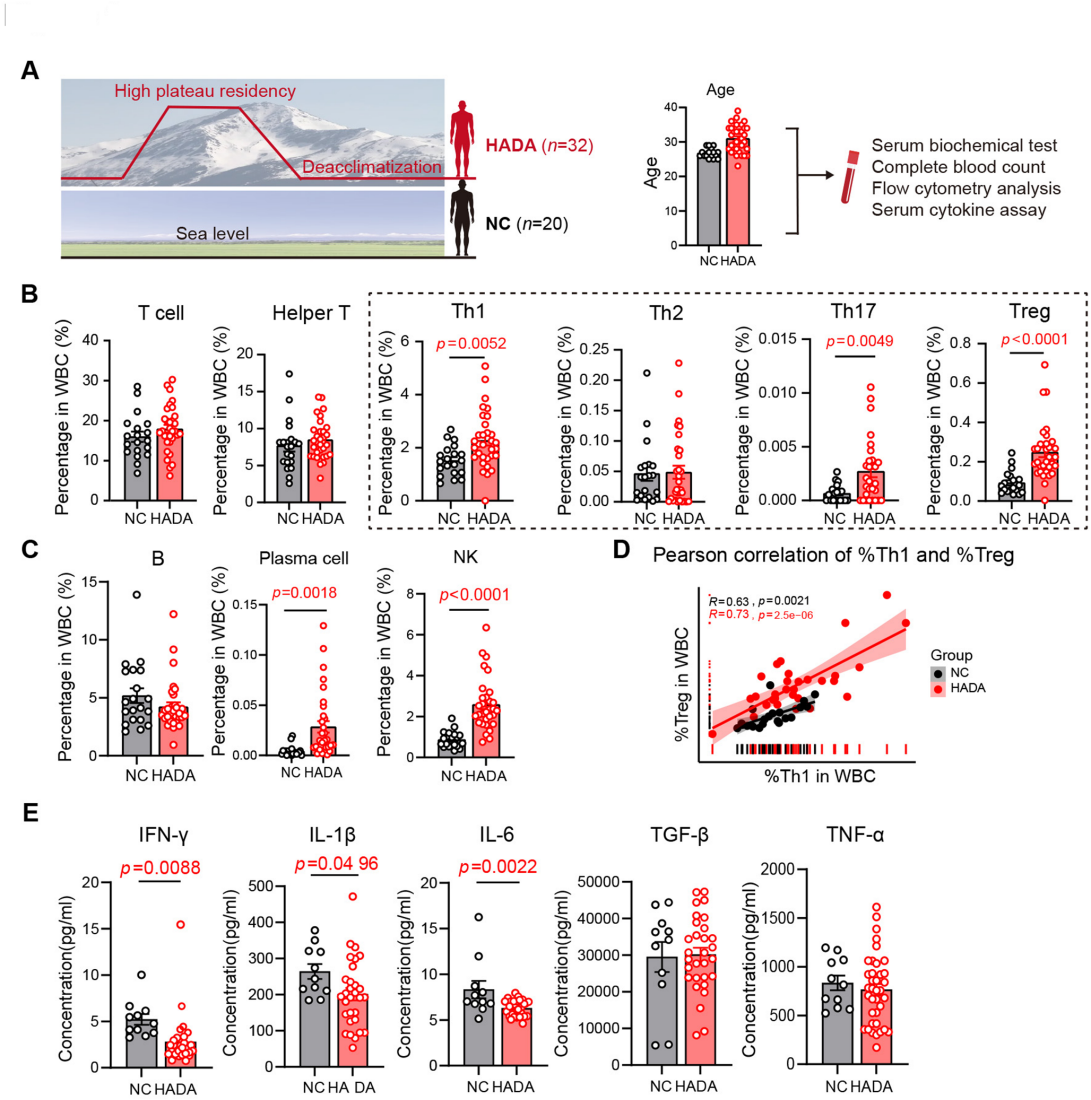
**Perturbation of peripheral immune populations in human during HADA. **(**A**) Overview of study design. Information for participants are described in detail in Methods and Materials. (**B**) Quantification of peripheral lymphocyte subsets, including T cells, helper T cells, Th1, Th2, Th17 and Tregs. Data are presented as mean ± SEM; statistical significance was determined by Student’s t-test. (**C**) Quantification of peripheral B cells, plasma cell and NK. Data are presented as mean ± SEM; statistical significance was determined by Student’s t-test. (**D**) The correlation of frequencies of Th1 and Treg cells in peripheral blood. (**E**) Plasma levels of cytokines in the peripheral blood from indicated groups. Data are presented as mean ± SEM; statistical significance was determined by Student’s t-test (*p<0.05, **p<0.01, ***p< 0.001, ****p< 0.0001)

A total of 32 individuals who migrated back to plains after a long-term high-altitude residence, termed as high-altitude de-acclimatization (HADA) group, and 20 altitude-naïve controls (NC) were included for analysis of peripheral immune cells (Figure 1A, Supplementary Figure 1A). Participants in the HADA group had a median altitude exposure of 4,000 meters with a median duration of 8 years at high altitude and a median of 1.5 years since returning to lowland environments. Participants in NC group had no history of high-altitude exposure (Supplementary Table 1). Peripheral blood samples from participants of both groups were collected for routine hematological analysis, followed by flow cytometry analyses.

Compared with those in NC group, individuals in the HADA group exhibited significantly reduced albumin-to-globulin (A:G) ratios and decreased globulin levels (Supplementary Table 2). Other biochemical indicators showed no significant differences between groups. Immunophenotypic profiling revealed slightly increase in the percentage of T cells in the peripheral white blood cells (WBCs) of HADA participants (Figure 1B). Elevated frequencies of T helper 1 cells (Th1, CD3⁺CD4⁺CXCR3⁺CCR6⁻) and Th17 cells (CD3+CD4+CCR6+CD161+) along with increased ratio of natural killer cells (NK, CD45+CD56+) and plasma cells (PC, CD19+IgD-CD27+CD38+CD138+) were detected (Figure 1B-C, Supplementary Figure 1B-E). The regulatory T cells (Tregs, CD3⁺CD4⁺CD25⁺CD127⁻) in the HADA group were also increased to over two folds (Figure 1B). These data indicated the peripheral immune disturbances caused by high-altitude de-acclimatization.

The synchronous increase of Th1/Th17/NK and Tregs was intriguing. The ratio of Tregs and Th1 showed highly positively correlation both in HADA and NC individuals (Figure 1D). The analysis of circulating cytokines showed a reduction in interferon-γ (IFN-γ) and interleukin-1β (IL-1β) levels in HADA individuals, whereas levels of transforming growth factor-β (TGF-β), tumor necrosis factor-α (TNF-α), and interleukin-6 (IL-6) displayed no significant differences between groups (Figure 1E), indicating an overall suppressed immune activity in HADA individuals.

### HADA induces increased peripheral Treg in both human and mice

**Fig. 2:**
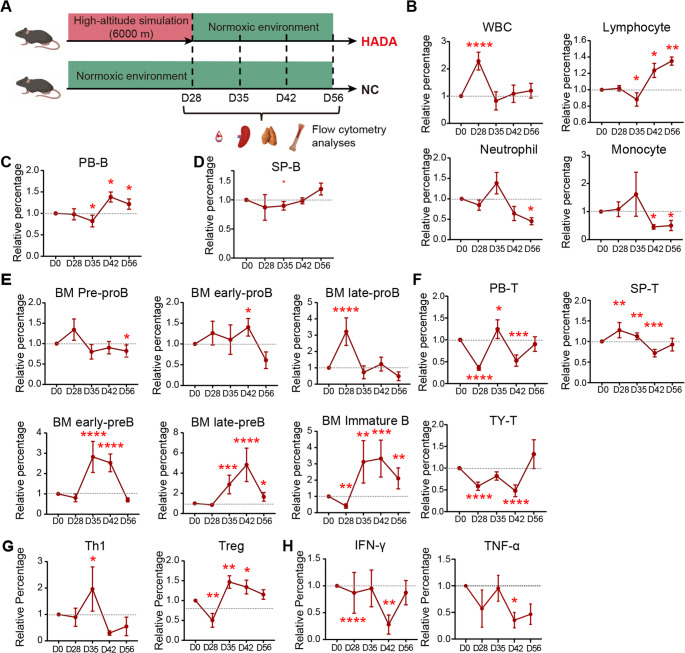
**Dynamic of immune remodeling during HADA in mice. ** (**A**) Experimental design showing strategy for HADA mice model construction. (**B**) Peripheral leukocyte composition, including total white blood cells, lymphocytes, neutrophils, and monocytes. The percentages are shown as relative values to normoxic controls. (**C**) Dynamics of proportion of B cells in peripheral blood. (**D**) Dynamics of proportion of B cells in the spleen. (**E**) Dynamics of proportion of B cells in the bone marrow. (**F**) Dynamics showing changes in T-cell populations in peripheral blood, spleen, and thymus across different stages. (**G**) Dynamics of percentages of Th1 and Treg cells in peripheral blood. (**H**) Dynamics of pro-inflammatory cytokines in the peripheral blood. Statistical significance was determined by Student’s t-test (*p < 0.05, **p < 0.01, ***p < 0.001, ****p < 0.0001)

To establish the HADA model, the mice were housed in a hypobaric hypoxic chamber simulating the oxygen concentration at an altitude of 6,000 m for 4 weeks followed by the transfer to normoxic conditions for another 4 weeks (Fig. 2A). The immune cells in peripheral blood, spleen, thymus and bone marrow of HADA mice and NC mice (consistently housed in normoxic condition) were traced. At day 28 within the chamber, the WBC concentration in the blood of HADA mice was dramatically increased with no significant alteration of lymphocyte, neutrophil and monocyte ratios observed (Fig. 2B). This could be caused by reduced water intake of HADA mice. During the later stage of de-acclimatization phase, the ratio of lymphocytes showed step-by-step increase while those of neutrophils and monocytes were declined (Fig. 2B), potentially reflecting a redistribution of innate immune components or a relative suppression of innate responses.

Flow cytometric analyses were performed to assess dynamics of hematopoietic lineages. The percentage of peripheral B cells was slightly decreased in day 35 and increased post day 42, while the number of B cells in spleen unperturbed (Fig. 2C-D), indicating that reoxygenation induced the expansion of B cells over extended timeframe. Further analysis of B cell populations in bone marrow showed that the B lineage differentiation in bone marrow was blocked by hypoxia at the stage of late-pro B cell (Fig. 2E), which was recovered once the mice were transferred into normoxic environment.

The percentage of T cells in PB was reduced both at day 28 and 42 (Fig. 2F). The percentage of T cells in thymus and spleen showed synchronized changes with those in PB at day 42, indicating that the decrease in T cells during HADA was pervasive throughout the body (Fig. 2F). Different from human samples, the percentage of Th1 in mice PB showed transient increase at day 35 (Fig. 2G). The percentage of Treg showed sustained increase at the stages of reoxygenation which was consistent with the observed characteristics in human PB (Fig. 1C). Most importantly, the levels of pro-inflammatory cytokines, TNF-α and IFN-γ, were significantly reduced at day 42 (Fig. 2H). Collectively, these findings demonstrated that the history of prolonged exposure to high-altitude hypoxia interrupted the immune populations.

### Peripheral Tregs experiencing HADA upregulate proliferation and immune-suppressing capacity

**Fig. 3:**
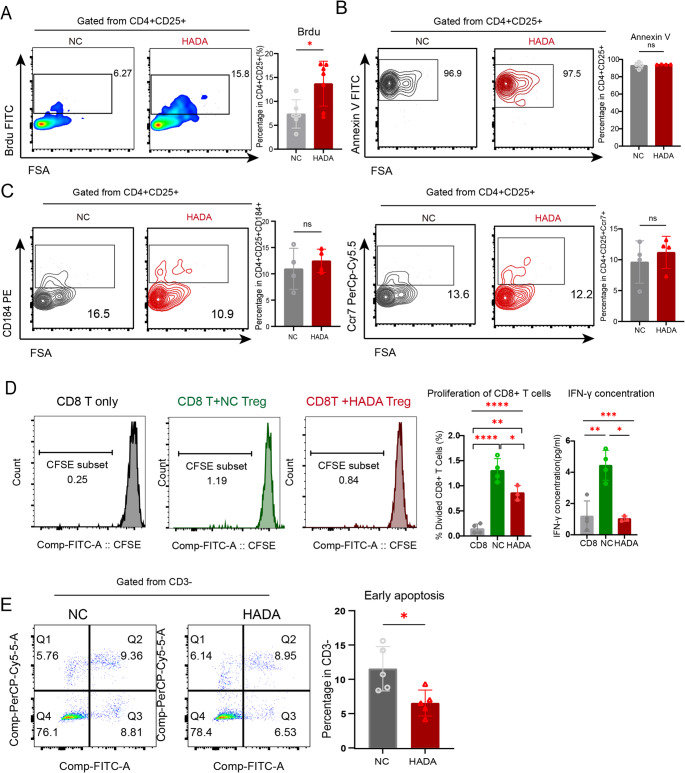
**Functional alterations of peripheral Treg cells at HADA phase.** (**A**) Proliferation of peripheral Treg cells assessed by flow cytometry. (**B**) Apoptosis levels of Treg cells compared between groups. (**C**) Chemotactic profiles of Treg cells analyzed by CXCR4 (CD184) and CCR7 expression. (**D**) Suppressive function of peripheral Treg cells evaluated by co-culture with CD8⁺ T cells, showing CD8⁺ T cell proliferation and IFN-γ production in culture supernatants. (**E**) Apoptosis of B16 after co-culture with CD3^+^ T cells, comparing groups with different Treg backgrounds. Data are presented as mean ± SEM; statistical significance was determined by Student’s t-test (*p < 0.05, **p < 0.01, ***p < 0.001, ****p < 0.0001)

To investigate the underlying cause of increased Treg cell numbers, the proliferating, apoptotic, migrating statuses of Tregs in PB were analyzed. Staining of BrdU showed increased proliferating proportion in Tregs from PB (Fig. 3A). No differences were observed in the apoptotic fractions in Tregs, as indicated by staining of Annexin V and Propidium Iodide (Fig. 3B). The expression of CCR7 and CXCR4 in Tregs was also not influenced by the experience of hypoxia (Fig. 3C), indicating that the origin of increased Tregs was likely not from spleen or other secondary lymphoid organ, though the proportion of Tregs in spleen was reduced (Supplementary Fig. 1B-E).

We subsequently evaluated the immune repressing function of Tregs in HADA mice. Tregs from peripheral blood were co-cultured with stimulated CD8^+^ T cells for 72 h. The proliferation rate of CD8 T cells were reduced by the addition of Tregs. However, the proliferation of CD8 T cells was further suppressed by Tregs from HADA mice (Fig. 3D). The concentration of IFN-γ in the supernatant further emphasized the upregulated immune-suppression function of Tregs in HADA mice (Fig. 3D).

For long-term effects of increased Tregs in HADA phase, T cells isolated from the peripheral blood of HADA and NC mice were co-cultured with B16 melanoma cells in vitro. Under 1:1 effector-to-target ratio, T cells from HADA mice increased the apoptotic ratio in B16 melanoma cells when compared to those from control group (Fig. 3E), indicating a suppressed function of whole T cells. These data along with the increased number of Tregs strongly suggested the role of Treg in suppressed immunity caused by HADA.

### Tregs experiencing HADA are changed at both transcriptomic and chromatin-accessibility level

**Fig. 4:**
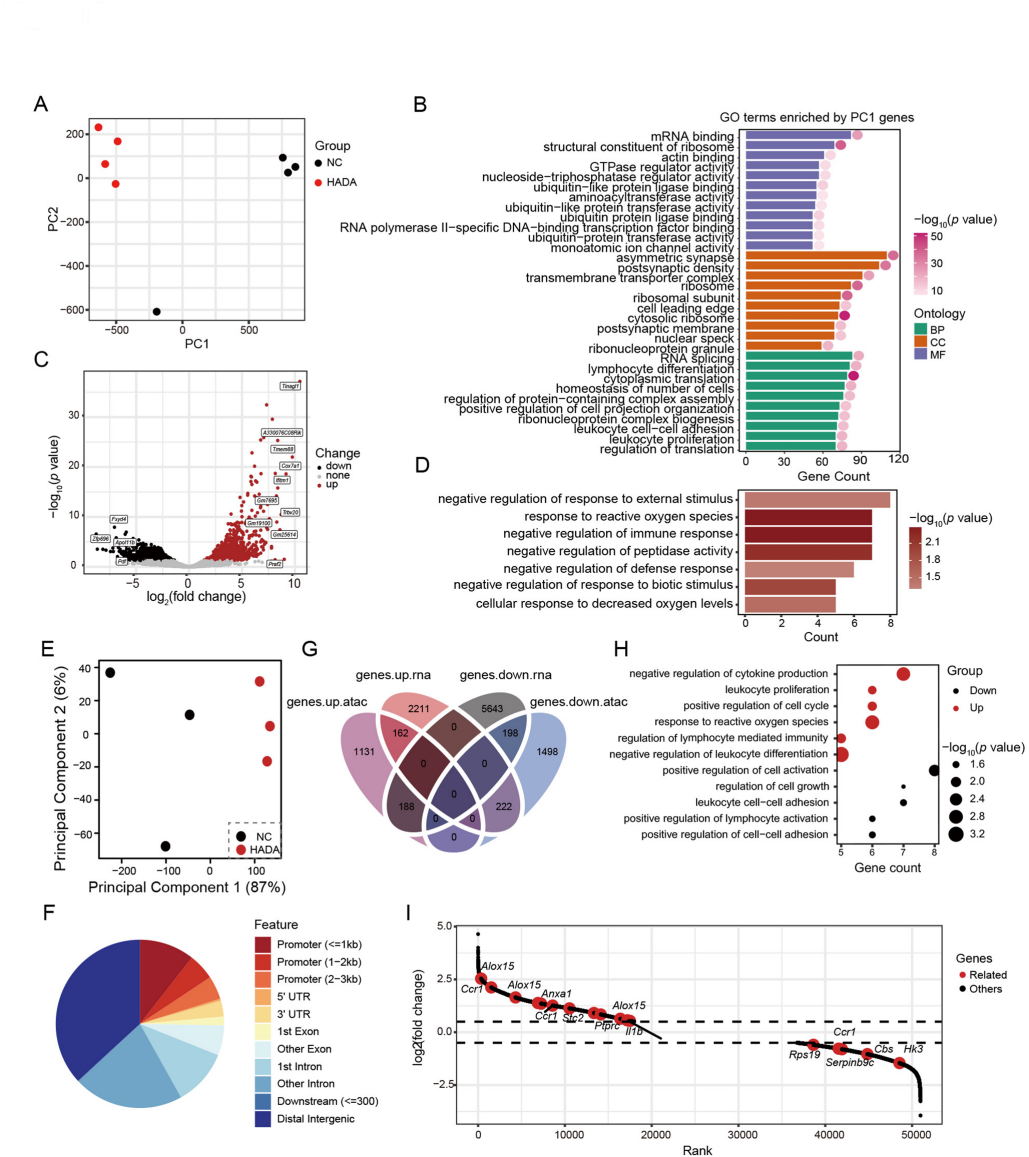
**Transcriptomic and ATAC profiling of peripheral Treg cells at HADA phase. **(**A**) Principal component analysis of peripheral Treg RNA-seq data. (**B**) GO terms enriched by genes contributing to principal component 1. (**C**) Presentation of differentially expressed genes in peripheral Treg, genes in red are those upregulated in Tregs from Hypo group. (**D**) Biological process GO terms enriched by genes upregulated by hypoxia treatment in peripheral Tregs. (**E**) PCA plot of ATAC-seq samples. (**F**) Pie plot showing the distribution of increased opened regions among groups. (**G**) Venn plot showing number of differentially expressed genes intersected across RNA-seq and ATAC-seq data. (**H**) GO terms enriched by genes mutually upregulated or downregulated at transcriptomic and ATAC-seq level. (**I**) Fold changes of ATAC-seq signal of genes contributed to upregulated features depicted in (**H**)

To decipher the molecular alterations in Tregs underlying their increased number and modified functions, RNA-seq was performed to FACS-sorted Tregs. Each group contains four samples. Tregs from two groups showed separated distribution in PCA plot with principal component 1 (PC1) accounting for the main differences (Fig. 4A). PC1 was related to translation, protein modification, lymphocyte differentiation and leukocyte proliferation (Fig. 4B). Compared to Control Tregs, HADA Tregs upregulated 1415 genes which enriched features related to negative regulation of response to external stimulus, response to reactive oxygen species, negative regulation of immune response and cellular response to decreased oxygen levels (Fig. 4C-D). These features illustrated that Tregs responded to decreased oxygen level and upregulated immune-suppressing function, which is accordant with our functional assay.

Due to the rare number of Tregs in PB, the chromatin accessibility data of peripheral blood mononuclear cells (PBMC) from two groups were obtained through ATAC-seq. The PCA plot showed obvious distinctions in chromatin accessibility, with PC1 represented the main differences of Treg features, indicating that HADA leads to alterations not only on transcriptional level but also on epigenomic level (Fig. 4E). About 20% of increased open frame of chromatin in PBMC was around the promoter region (Fig. 4F).

To focus on the epigenomic regulation caused by HADA in Tregs, the DEGs obtained from ATAC-seq and RNA-seq were intersected. Totally, 162 genes transcriptionally upregulated by Tregs showed upregulated chromatin accessibility in PBMC ATAC-seq (Fig. 4G). These genes enriched features related to negative regulation of cytokine production, leukocyte proliferation, positive regulation of cell cycle and negative regulation of leukocyte differentiation (Fig. 4H). *Alox15*,* Il1b* and *Ccr1* were among these genes (Fig. 4I). Correspondingly, a total of 198 genes downregulated transcriptionally by Tregs showed downregulated chromatin accessibility in PBMCs. These genes enriched features related to positive regulation of immune processes (Fig. 4H). These data strongly suggested that upregulated proliferation and enhanced immune-suppressing feature in Tregs were under the regulation of chromatin accessibility.

### Nrf2 mediates the molecular response of Tregs to HADA

**Fig. 5:**
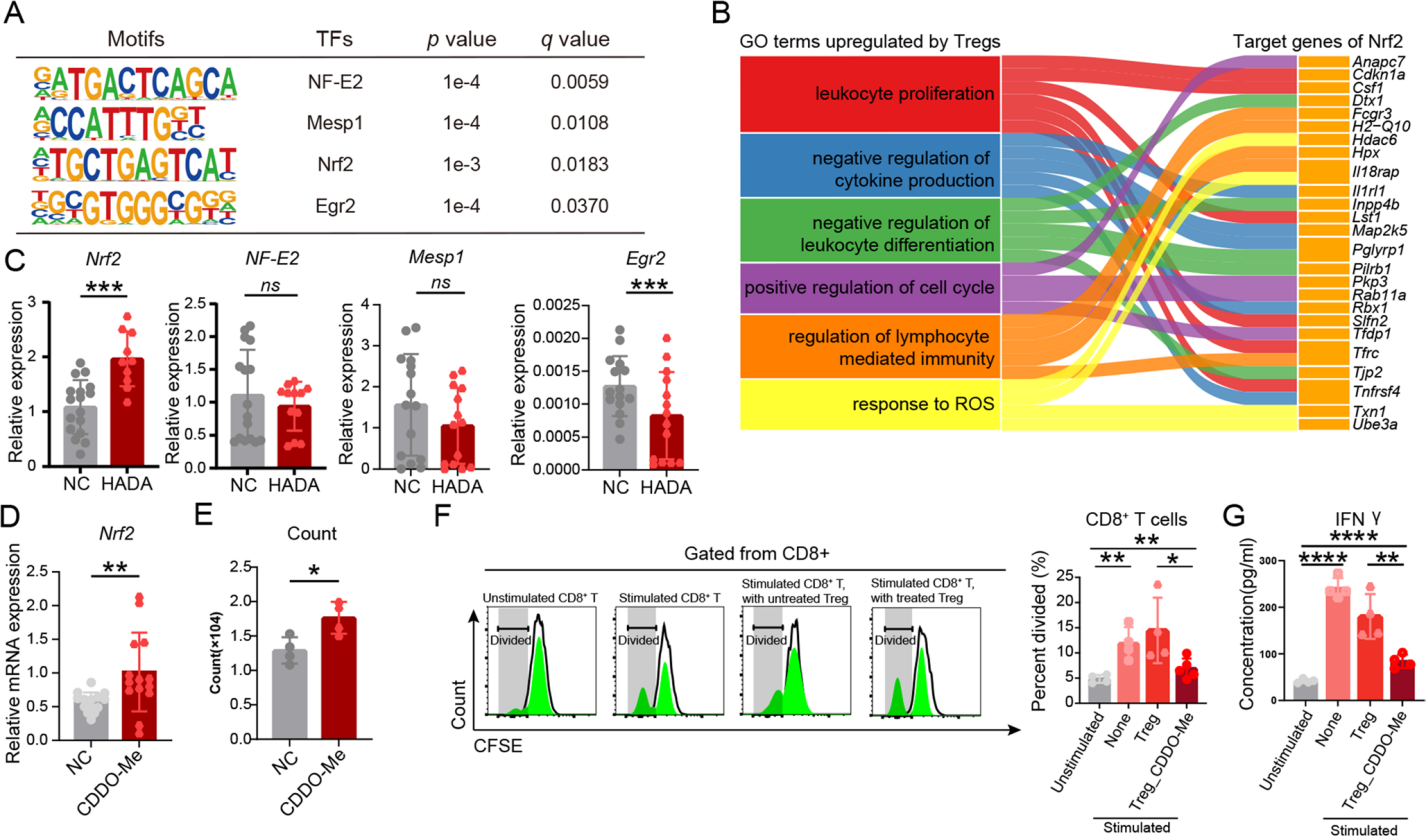
**Screening of transcription factor modulating Treg alterations in HADA.** (**A**) Motifs and transcription factors predicted for HADA-induced change in Tregs. (**B**) Bar plots showing expression of candidate transcription factors in Tregs. (**C**) Relations among upregulated features and target genes predicted to be regulated by Nrf2. (**D**) Expression of Nrf2 in Tregs upon CDDO-Me stimulation. (**E**) Number of Tregs after CDDO-Me stimulation. (**F**) FACS analysis of CD8^+^ T division using CFSE staining. (**G**) The level of INF-γ secreted by CD8^+^ T cells in indicated groups. Data are presented as mean ± SEM; statistical significance was determined by Student’s t-test or one-way analysis of variance (ANOVA) (*p < 0.05, **p < 0.01, ***p < 0.001, ****p < 0.0001)

The transcription factors (TFs) binding to 162 genes both upregulated in transcriptomic data and ATAC data were predicted (Fig. 5A). Among the four TFs screened out, Nuclear Factor Erythroid 2 (*NF-E2*) was reported to regulate the differentiation of megakaryocytes and erythroid cells [22]. Mesoderm Posterior 1 (*mesp1*) is the primary modulator of fate priming of mesoderm and no reports on its regulation in immunity has been published. Nuclear Factor Erythroid 2-related factor 2 (*Nrf2*) is involved in anti-oxidative, anti-inflammatory and anti-toxicity processes [23]. It has been reported that the upregulation of Nrf2/HO-1 signals induced the generation of Treg cells and release the levels of IL-6, IL-1β and TNF-α [24] which was probably due to its strong function against oxidative stress. Early Growth Response 2 (*Egr2*) is the main regulator of peripheral nervous system and is related to immune suppressing function of Tregs [25].

The expression of TFs enrolled by ATAC-seq data was analyzed in Tregs from PB. The expression of *Nrf2* was significantly upregulated in Tregs from HADA mice (Fig. 5C). The molecules downstream of the transcriptomic regulation of *Nrf2* were subsequently predicted based on the regulatory motifs. Among the 173 targets of *Nrf2*, 29 genes contributed to features related to negative regulation of cytokine production, positive regulation of cell cycle, response to reactive oxygen species and regulation of lymphocyte-mediated immunity (Fig. 5B). This confirmed the regulatory role of *Nrf2* in Treg response to HADA.

To verify the role of *Nrf2* in HADA-induced alterations of Treg cells, peripheral blood Treg cells were treated with *Nrf2* agonist Bardoxolone methyl (CDDO-Me) for 24 h to induce the overexpression of *Nrf2*. The upregulation of *Nrf2* was verified first in Tregs (Fig. 5D). After further culture for 48 h, Tregs treated with CDDO-Me experienced a higher level of expansion (Fig. 5E). The immune-suppressing capacity was further evaluated based on co-culture with stimulated CD8^+^ T cells for another 72 h. The expansion of CD8^+^ T cells was significantly inhibited by Tregs treated with CDDO-Me (Fig. 5F). The concentration of IFN-γ was also reduced (Fig. 5G). This along with previous data indicated that Nrf2 serves as a partial, if not all, mechanistic explanation for the effects of HADA on Treg cells.

## Discussion

The long-term responses of individuals during HADA phase have not yet been fully characterized. We systematically investigated immune alterations in both human subjects and mouse models that experienced high-altitude exposure or hypoxic conditions followed by a return to low-altitude or normoxic environments. Our results revealed a consistent increase in Tregs in the peripheral blood of both humans and mice undergoing HADA. Notably, the expanded Treg population exhibited enhanced proliferative capacity and immunosuppressive function, which was associated with reduced immune activity against cancer cells. Through integrated RNA sequencing of Tregs and ATAC sequencing of peripheral white blood cells, we identified *Nrf2*, a well-established regulator of cellular redox homeostasis, as a key transcription factor modulating Treg abundance and function during HADA. Together, these findings provide the first comprehensive evidence of long-term immune remodeling induced by HADA and offer important insights into the immunological consequences of both high-altitude acclimatization and de-acclimatization. By delineating immune alterations associated with HADA in both human cohorts and animal models, this study enables the identification and monitoring of populations susceptible to immune dysregulation during the de-acclimatization period. Furthermore, targeting *Nrf2* through preventive pharmacological interventions may represent a promising strategy to mitigate HADA-associated immune suppression, thereby supporting a paradigm shift from post-onset treatment toward predictive, preventive, and personalized medicine (3PM/PPPM) in the management of high-altitude de-acclimatization–related health risks.

The long-term consequences of a sustained increase in Treg activity and a resultant state of immunosuppression present a significant clinical paradox. While Tregs are indispensable for maintaining self-tolerance and preventing autoimmunity, their chronic overabundance can severely compromise immunosurveillance [26]. This impaired immune vigilance creates a permissive environment for the emergence and progression of malignancies, as nascent tumor cells are more likely to evade detection and destruction [26]. Furthermore, it heightens susceptibility to opportunistic infections and viral reactivation, as seen with latent herpesviruses like Epstein-Barr virus or cytomegalovirus [27]. From a PPPM perspective, these observations underscore the importance of early identification of individuals at risk of Treg-dominated immune imbalance, enabling predictive risk stratification, targeted preventive interventions, and personalized immune modulation strategies. Maintaining a finely tuned immune homeostasis is therefore not only essential for immune tolerance but also represents a key determinant of long-term disease susceptibility and health outcomes.

The observed increase in regulatory T cells (Tregs) within the peripheral blood likely originates from two primary mechanisms: thymic derivation and peripheral induction/expansion [28]. The thymus serves as the primary source, generating a continuous output of natural Tregs (nTregs) that are inherently programmed for immunosuppressive function; an upregulation in thymic Treg production could directly contribute to elevated peripheral counts [28]. However, a significant proportion of the expanded Treg pool is probably acquired in the periphery. Induced Tregs (iTregs) can differentiate from conventional CD4^+^ T cells in secondary lymphoid organs, such as lymph nodes and the spleen, particularly under specific cytokine milieus rich in TGF-β and interleukin-2 (IL-2) [29]. Furthermore, chronic antigenic stimulation at sites of inflammation, such as in autoimmune conditions, tumors, or persistent infections, can act as a crucial niche for the local expansion and conversion of Tregs, which subsequently migrate into the circulation. Thus, the periphery acts not merely as a reservoir but as an active factory, where both the *de novo* generation and clonal amplification of Tregs can occur, driven by local immunological cues.

The acclimatization to hypoxia and the subsequent return to normoxia may initiate a complex interplay between oxidative stress and immune regulation, potentially centered on the Nrf2 pathway [30]. Upon descent, the reoxygenation is thought to induce a significant burst of reactive oxygen species (ROS), creating a state of oxidative stress [31]. This oxidative environment can have a dual effect: it actively triggers the activation of the transcription factor Nrf2, the master regulator of antioxidant response elements (ARE), which upregulates cytoprotective genes to restore redox homeostasis [32]. Concurrently, elevated ROS and the subsequent cellular stress can act as a potent signaling cue for the immune system [33]. We hypothesized that this ROS-Nrf2 axis may create an anti-inflammatory milieu conducive to the induction and functional enhancement of Tregs. Thus, the post-descent surge in oxidative stress may represent a pivotal regulatory trigger that couples metabolic reprogramming with immune tolerance via Nrf2-dependent Treg expansion during high-altitude de-acclimatization. This integrative mechanism offers a biologically grounded framework for early identification of immune vulnerability, longitudinal immune monitoring, and stratification of individuals at risk for prolonged immunosuppression. Importantly, targeting this regulatory axis opens new avenues for preventive intervention and individualized immune rebalancing, thereby facilitating a transition from reactive management toward predictive, preventive, and personalized medicine (PPPM) in the context of HADA.

### Limitations

Despite the encouraging findings of this study, several limitations should be acknowledged. First, although an increase in peripheral blood Treg cells was consistently confirmed in both human cohorts and animal models, the sample size of the human population remains limited and should be expanded to obtain more generalizable conclusions. Second, the involvement of Nrf2 as a key molecular target mediating Treg-associated immunosuppressive functions requires further systematic evaluation in individuals undergoing HADA to extend our findings. Finally, although Nrf2 was identified as a central focus in this study, broader exploration of therapeutic strategies targeting this molecule is still needed in future investigations.

### Conclusion, expert recommendations, and outlook in the framework of 3PM

In summary, our findings collectively delineate the landscape of peripheral immune cell alterations during the HADA phase in both humans and mice. We demonstrate that the expansion and heightened immunosuppressive function of Treg cells drive an overall immunosuppressive state, which likely underpins the clinical manifestations of immune dysregulation upon returning to lower altitude. Furthermore, multi-omic analyses pinpointed Nrf2 as a pivotal upstream regulator of this process, offering a mechanistic foundation for developing targeted therapeutic strategies.

Effective management of HADA within the PPPM framework requires coordinated efforts across predictive, preventive, and personalized strategies. From a predictive perspective, priority should be given to monitoring dynamic changes in circulating Treg cell populations in individuals returning to normoxic environments after prolonged hypoxic exposure. Routine surveillance, systematic assessment of immune status, and long-term follow-up are essential to identify sustained immunosuppression at an early stage and to prevent irreversible consequences, including chronic inflammation and increased susceptibility to malignancies. Preventive strategies should emphasize biomarker-guided, individualized nutritional interventions and lifestyle adjustments tailored to an individual’s physiological condition. In this context, mitochondrial quality control plays a critical role. Hypoxic exposure at high altitude challenges mitochondrial function, leading to reduced mitochondrial density and altered energy metabolism, which contribute to altitude-related disorders (AMS) [34]. Importantly, even after descent and restoration of normoxia, mitochondrial recovery may remain incomplete; therefore, post-descent assessment of mitochondrial function is warranted. Targeted nutritional strategies may be implemented to support mitochondrial health and functional restoration [35, 36]. Personalized medicine approaches can be further refined by integrating multi-omics data with intracellular *Nrf2* expression profiles, enabling individualized intervention plans and dynamic health management. Moreover, in-depth elucidation of Nrf2-associated molecular mechanisms will facilitate the development of more precise targeted prevention strategies and personalized medical services for HADA. Overall, these recommendations aim to mitigate immunosuppressive states in individuals with HADA, prevent irreversible outcomes driven by immune dysregulation, and ultimately improve long-term health and quality of life.

## Supplementary Information

Below is the link to the electronic supplementary material.

Supplementary figure 6 Supplementary Material 1 (PNG 710 KB)

High Resolution Image (tif 12.2 MB)

Supplementary figure 7 Supplementary Material 2 (PNG 364 KB)

High Resolution Image (tif 9.34 MB)

Supplementary Material 3 (xlsx 9.41 KB)

Supplementary Material 4 (xlsx 11.4 KB)

## Data Availability

The raw data of RNA-seq and ATAC-seq generated in our study have been deposited in the Gene Expression Omnibus (GEO) under the accession number GSE315734 and GSE315544.

## Abbreviations

HADA: High altittude de-acclimatization
Nrf2: Nuclear factor erythroid 2–related factor 2
Treg: Regulatory T cells
RNA: Ribonucleic acid
ATAC: Assay for transposase-accessible chromatin
HADAS: High-altitude de-acclimatization syndrome
HIFs: Hypoxia-inducible factors
JAK-STAT: Janus kinase–signal transducer and activator of transcription
NOD: Nucleotide-binding oligomerization domain
IL: Interleukin
TNF: Tumor necrosis factor
SPF: Specific pathogen-free
ELISA: Enzyme linked immunosorbent assay
IFN: Interferon
TGF: Transforming growth factor
BCA: Bicinchoninic acid
FBS: Fetal bovine serum
Foxp3: Forkhead box P3
Th: T helper
BrdU: 5-Bromo-2′-deoxyuridine
RPMI: Roswell park memorial institute
CFSE: Carboxyfluorescein succinimidyl ester
cDNA: Complementary DNA
PCR: Polymerase chain reaction
FASTQ: FASTA with quality scores
DEGs: Differentially expressed genes
GO: Gene ontology
NC: Altitude-naïve controls
WBC: White blood cell
NK cell: Natural killer cell
PC: Plasma cell
PB: Peripheral blood
CCR7: C-C chemokine receptor type 7
CXCR4: C-X-C chemokine receptor type 4
PCA: Principal component analysis
PBMC: Peripheral blood mononuclear cells
Alox15: Arachidonate 15-lipoxygenase
Il1b: Interleukin 1 beta
Ccr1: C-C chemokine receptor type 1
TFs: Transcription factors
AMS: Altitude-related disorders
NF-E2: Nuclear factor erythroid 2
mesp1: Mesoderm posterior 1
Egr2: Early growth response 2
CDDO-Me: Bardoxolone methyl
ROS: Reactive oxygen species
HA: High altitude
ARE: Antioxidant response elements
STAR aligner: Spliced transcripts alignment to a reference
FACS: Fluorescence-activated cell sorting
