# Supplementary figures and images for: High-altitude de-acclimatization and long-term immune suppression: the role of Nrf2 in Treg function targeting 3PM
Source: EPMA J. 2026 Mar 4;17(1):57–71. doi: 10.1007/s13167-026-00442-x (PMC12976330; doi:10.1007/s13167-026-00442-x)

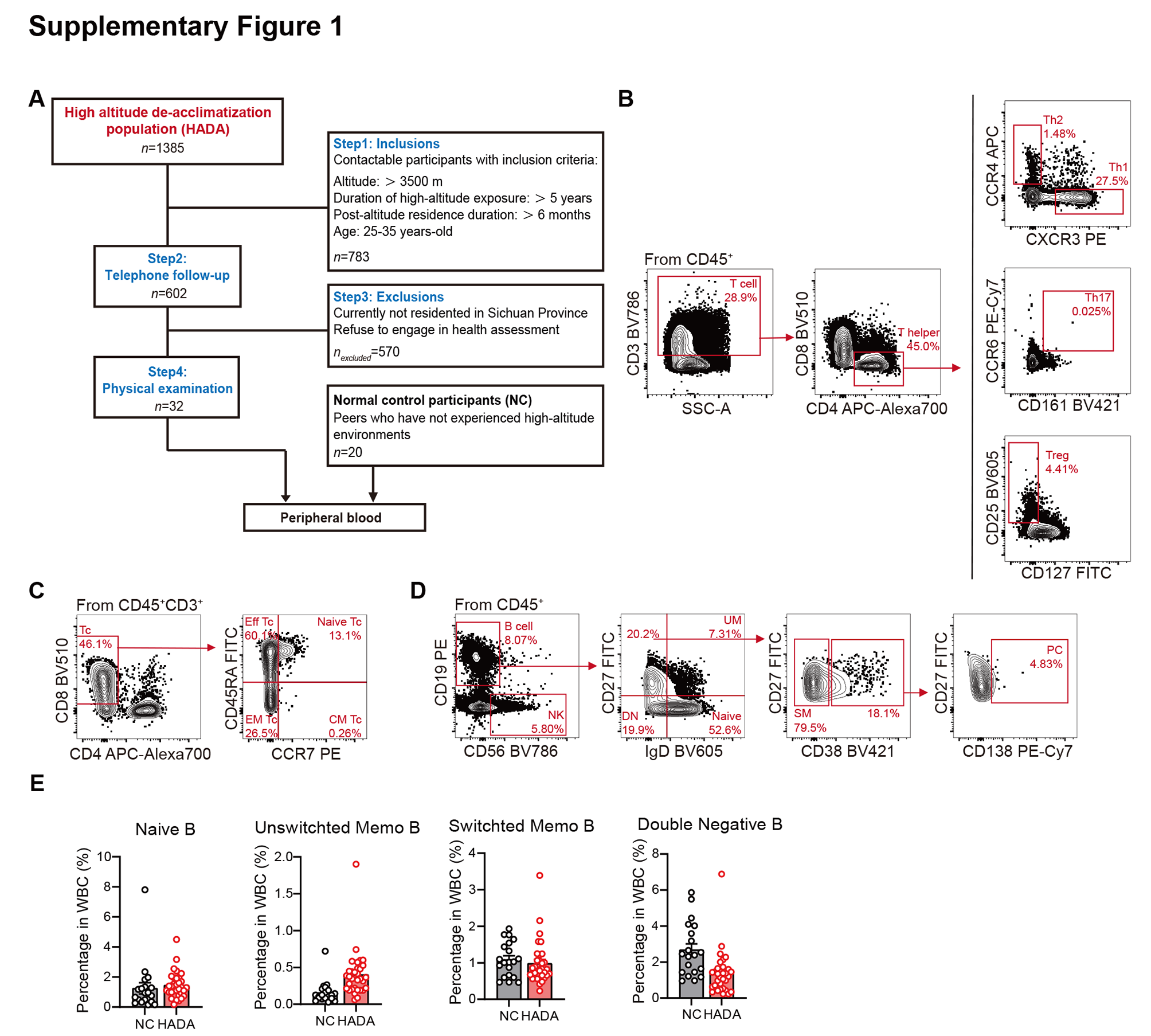

Supplement: Supplementary file 1 — Supplementary Material 1 (PNG 710 KB) [file 13167_2026_442_Fig6_ESM.png]

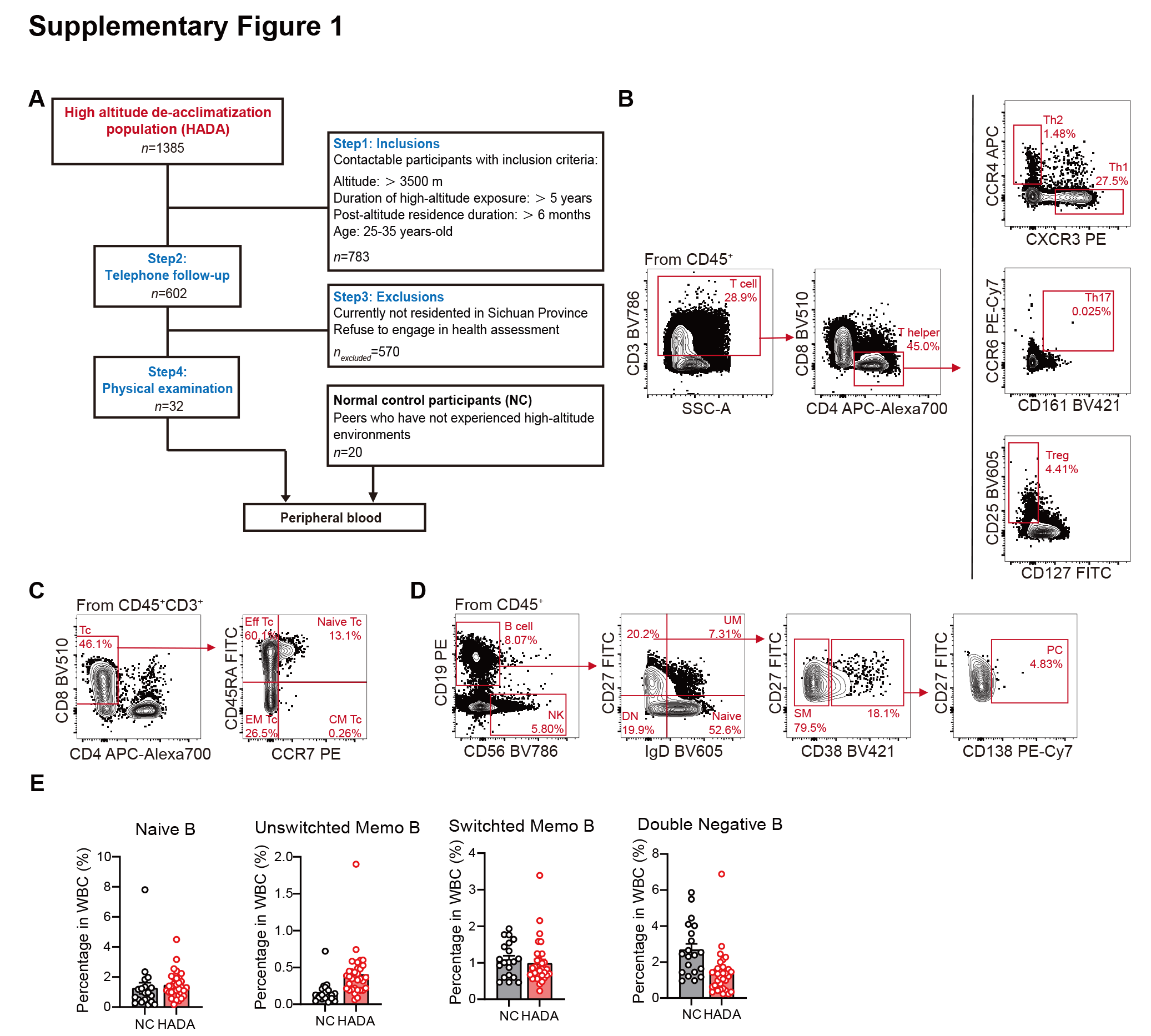

Supplement: Supplementary file 2 — High Resolution Image (tif 12.2 MB) [file 13167_2026_442_MOESM3_ESM.tif]

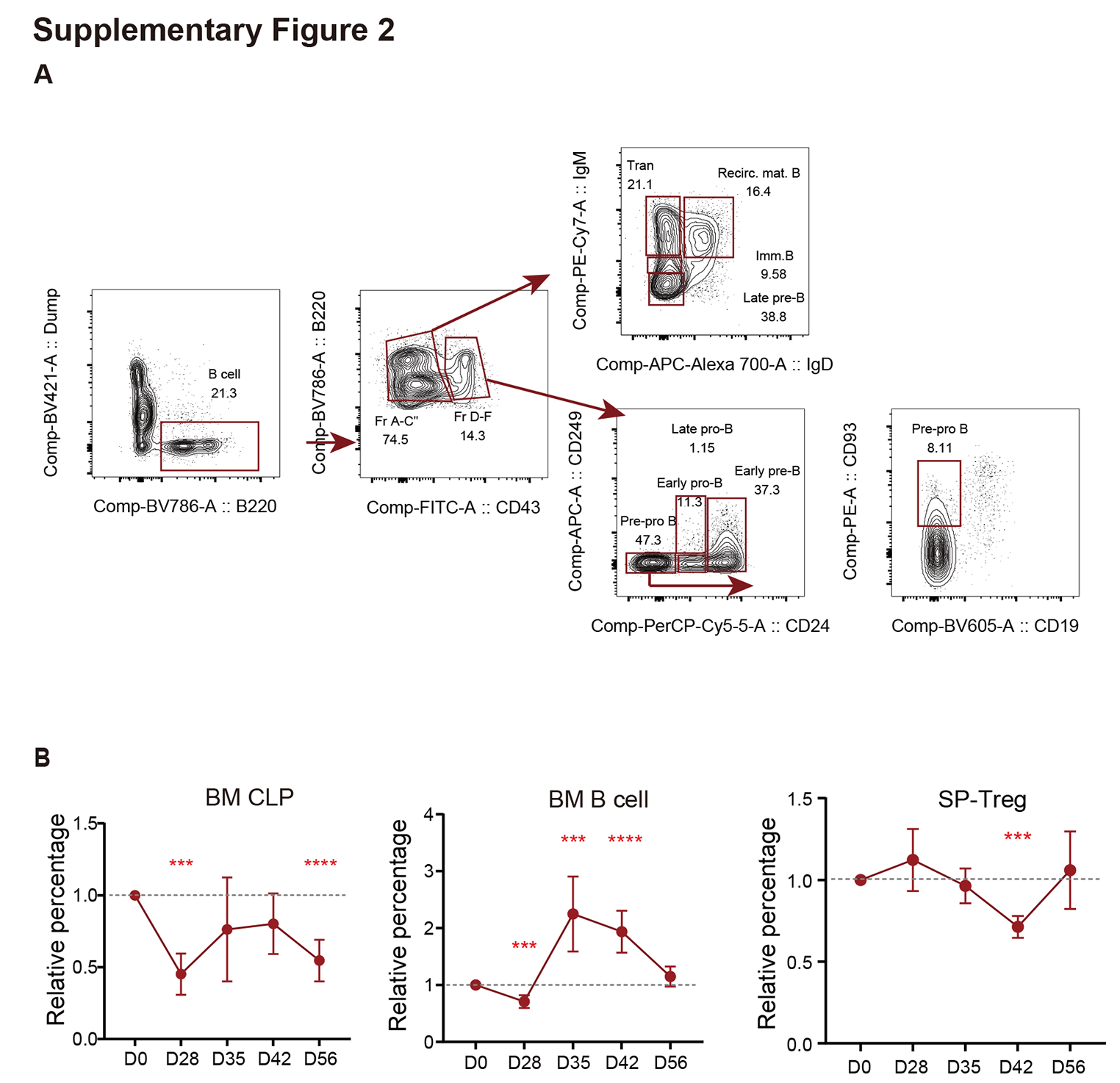

Supplement: Supplementary file 3 — Supplementary Material 2 (PNG 364 KB) [file 13167_2026_442_Fig7_ESM.png]

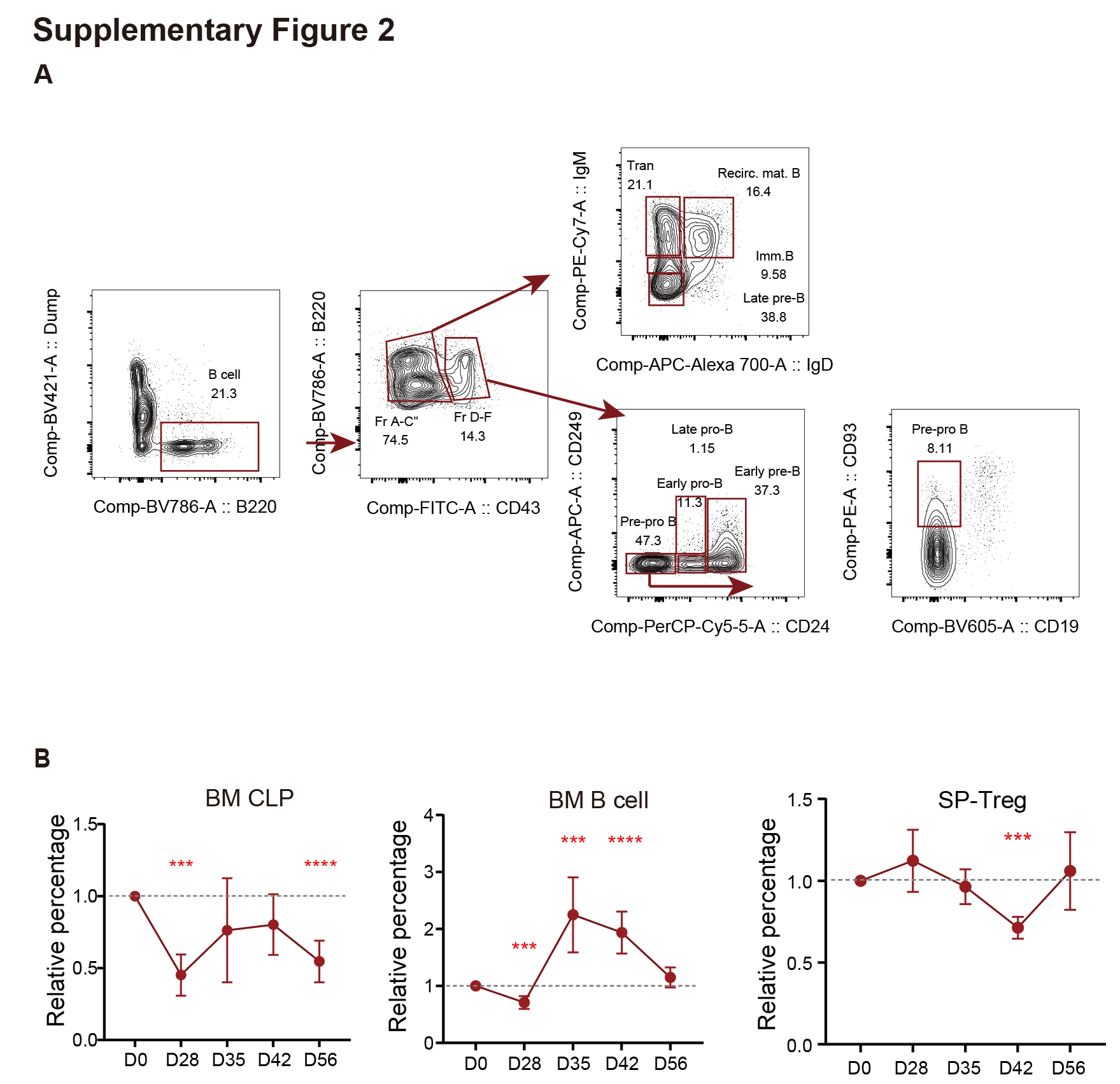

Supplement: Supplementary file 4 — High Resolution Image (tif 9.34 MB) [file 13167_2026_442_MOESM4_ESM.tif]
